# Prevalence of sarcopenia in inpatients 70 years and older using different diagnostic criteria

**DOI:** 10.1002/nop2.219

**Published:** 2018-11-28

**Authors:** Esmee M. Reijnierse, Angela Buljan, Camilla S. L. Tuttle, Jeanine van Ancum, Sjors Verlaan, Carel G. M. Meskers, Andrea B. Maier

**Affiliations:** ^1^ Department of Medicine and Aged Care, @AgeMelbourne The Royal Melbourne Hospital, The University of Melbourne Melbourne Victoria Australia; ^2^ Faculty of Behavioural and Movement Sciences, Department of Human Movement Sciences, @AgeAmsterdam Vrije Universiteit Amsterdam, Amsterdam Movement Sciences Amsterdam The Netherlands; ^3^ Department of Internal Medicine, Section of Gerontology and Geriatrics VU University Medical Center Amsterdam The Netherlands; ^4^ Department of Rehabilitation Medicine VU University Medical Center Amsterdam The Netherlands

**Keywords:** aged, hospitalization, muscle, muscle mass, muscle strength, sarcopenia

## Abstract

**Aim:**

To compare prevalence rates of sarcopenia applying multiple diagnostic criteria in hospitalized older patients.

**Design:**

Observational, longitudinal EMPOWER study.

**Methods:**

A total of 378 hospitalized inpatients aged 70 years and older were recruited. Muscle mass and strength were measured using bioelectrical impedance analysis and handheld dynamometer respectively. Nine commonly used diagnostic criteria for sarcopenia were applied. Analyses were stratified for sex.

**Results:**

Mean age was 79.7 years (*SD* 6.43) and 50.8% were males. Depending on the applied criterion, prevalence of sarcopenia ranged between 12.0–75.9% in males and 3.1–75.3% in females. Males had a higher prevalence of sarcopenia compared with females in all but one of the applied diagnostic criteria. In males, highest prevalence of sarcopenia was found using muscle mass as diagnostic criterion while in females this was observed when using muscle strength. Five male and one female hospitalized older patients were sarcopenic according to all applied diagnostic criteria.

## 1 INTRODUCTION

Sarcopenia, characterized by generalized, progressive, age‐related low muscle mass and muscle strength (Rosenberg, 1997), is associated with risk of falls (Clynes et al., 2015), fractures (Clynes et al., 2015), morbidity (Janssen, Baumgartner, Ross, Rosenberg, & Roubenoff, 2004) and mortality (Beaudart, Zaaria, Pasleau, Reginster, & Bruyere, 2017; Volpato et al., 2014) in older populations. Identifying appropriate treatments for sarcopenia has been limited by the lack of consensus on diagnostic criteria for this disease (McLean & Kiel, 2015). Diagnostic criteria for sarcopenia consist of one or more measures of muscle mass, muscle strength and/or physical performance together with sex‐specific cut‐off points (Kim et al., 2016; Reijnierse et al., 2015).

Acute illness in conjunction with prolonged bed rest and low physical activity may increase the likelihood of experiencing adverse health outcomes in older acute inpatients (Bianchi et al., 2017). Early diagnosis of sarcopenia might therefore be essential to prevent further health decline in this population. Numerous proposed diagnostic criteria for sarcopenia use a measure of physical performance such as gait speed (Cruz‐Jentoft et al., 2010; Fielding et al., 2011; Studenski et al., 2014). However, in an older inpatient population, physical performance is frequently not possible to measure due to acute illness (Coker, Hays, Williams, Wolfe, & Evans, 2015; Janssen, Heymsfield, & Ross, 2002; Kortebein et al., 2008). Moreover, it has been shown that the prevalence of sarcopenia highly depends on the used diagnostic criteria in non‐hospitalized populations (Bijlsma et al., 2013; Reijnierse et al., 2015). The aim of the study is to compare prevalence rates of sarcopenia using nine commonly used diagnostic criteria in inpatients 70 years and older.

## 2 METHODS

### 2.1 Study design

The Evaluation of Muscle parameters in a Prospective cohort of Older patients at clinical Wards Exploring Relations with bed rest and malnutrition (EMPOWER) is an observational, prospective and longitudinal inception cohort. The EMPOWER study was conducted from April 2015 to December 2015 at a university hospital (VU University Medical Center, Amsterdam, The Netherlands). Over this period, 828 patients aged 70 years and older were admitted to the internal medicine, acute medicine, trauma and orthopaedic wards and considered eligible to participate in the study. Patients were excluded if they were nursed in air‐pressure isolation rooms, suffering from terminal illness, or expected to be discharged within 24 hr. All patients were required to be able and willing to provide signed informed consent. In total, 378 patients were included in the study. The study was reviewed and approved by the Medical Ethics Committee of the VU University Medical Center, Amsterdam, The Netherlands.

### 2.2 Patient characteristics

Medical records were reviewed for information about age, number of chronic medications, number of chronic diseases and length of stay. Current smoking (yes/no), current alcohol use (yes/no), living situation (living independently), risk of malnutrition (Short Nutritional Assessment Questionnaire—SNAQ), SNAQ‐score ≥2 at risk; Kruizenga, Seidell, de Vet, Wierdsma, & van Bokhorst–de van der Schueren, 2005), use of walking aid (yes/no), immobility for more than one week in the previous three months, self‐reported balance impairments and disabilities in daily life (Katz Index of Activities of Daily Living—ADL, Katz ADL‐score <2 indicating ADL dependency) were obtained at the bedside (Katz, Ford, Moskowitz, Jackson, & Jaffe, 1963).

### 2.3 Anthropometric measurements

Weight (kg) was measured using a weighting chair. An estimate for weight was obtained from the patient or his/her relative for bedbound patients. Height (cm) was estimated using knee height, as proposed by the Longitudinal Aging Study Amsterdam (LASA) formula (Male = 74.48 + [2.03 * knee height in cm]—(0.15 * age), female = 68.74 + [2.07 * knee height in cm]—[0.16 * age]). Body mass index (BMI) was expressed as kg/m^2^ (Chumlea, Roche, & Steinbaugh, 1985; LASA, 2018).

### 2.4 Muscle mass

Direct segmental multifrequency bioelectrical impedance analysis was used to measure muscle mass (DSM‐BIA; In Body S10; Biospace CO., Ltd, Seoul, Korea). DSM‐BIA is a validated method for estimating skeletal muscle mass (SMM) compared with dual‐energy X‐ray absorptiometry (Ling et al., 2011). Patients were measured in supine position. Their extremities had to be straightened not touching the core. During the measurement, patients were asked not to move. Fifty‐seven patients (34 males and 23 females) were excluded from DSM‐BIA measurements due to having a pacemaker, implantable cardioverter‐defibrillator, plaster or bandages at the place of the electrodes or amputated arm and/or leg. Measures of muscle mass were expressed as appendicular lean mass (ALM) divided by height squared, ALM (kg) divided by BMI, skeletal muscle mass (SMM in kilograms) as percentage of body weight and skeletal muscle index (SMI: SMM divided by height squared).

### 2.5 Muscle strength

Handgrip strength (HGS) was measured two times for each hand using the Jamar Hydraulic Handheld Dynamometer (Sammons Preston, Inc. Bolingbrook, IL, USA; Reijnierse et al., 2017). Measurements were obtained with patients in an upright position with their elbow flexed at 90° and the elbow unsupported. If patients were bedbound, HGS was measured in a semisupine position in an angle of approximately 30° with the elbow unsupported. Patients were asked to squeeze maximally with encouragement from the assessor. The maximum value obtained from either hand was used for analyses and expressed in kilograms.

### 2.6 Diagnostic criteria for sarcopenia

Nine sets of diagnostic criteria for sarcopenia were applied. Six of these definitions were single measure based: five included only muscle mass (Baumgartner et al., 1998; Delmonico et al., 2007; Janssen et al., 2004, 2002; Kelly, Wilson, & Heymsfield, 2009) and one only muscle strength (Lauretani et al., 2003). Three definitions were multiple measure based, that is, muscle mass, muscle strength and/or physical performance as proposed by different consensus working groups for sarcopenia including the European Working Group on Sarcopenia in Older People (EWGSOP), International Working Group on Sarcopenia (IWGS) and Foundation for the National Institutes of Health (FNIH) (Cruz‐Jentoft et al., 2010; Fielding et al., 2011; Studenski et al., 2014). The multiple measure‐based criteria included gait speed for physical performance, which was omitted from the definition in this study due to inability to perform gait speed at hospital admission.

### 2.7 Statistical analysis

Descriptive characteristics were presented as mean and standard deviation (*SD*) for data with a normal distribution. Skewed data were presented as median and interquartile range (IQR). Numbers and percentage (%) were used for categorical data. Analyses were stratified by sex.

Descriptive statistics were used to analyse the prevalence rates of sarcopenia according to the nine applied diagnostic criteria at admission. The overlap between the different diagnostic criteria at admission was visualized using a Venn diagram. Only individuals who were classified as sarcopenic according to at least one set of diagnostic criteria were included in the figures. Statistical analyses were performed using the Statistical Package for the Social Sciences (IBM SPSS Statistics for Windows, Version 24, IBM Corp, Armonk, NY).

## 3 RESULTS

### 3.1 Patient characteristics

Table 1 shows the characteristics of inpatients, stratified by sex. The population consisted of 186 females (mean age of 79.1 years; *SD* 6.28) and 192 males (mean age of 80.3 years; *SD* 6.54). Use of a walking aid and self‐reported balance impairments were more frequent in females compared with males. Males had higher muscle mass and handgrip strength compared with females.

**Table 1:** Descriptive characteristics of inpatients, stratified by sex

	Total (*N* = 378)	Males (*N* = 192)	Females (*N* = 186)
Age, years	79.7 (6.4)	79.1 (6.3)	80.3 (6.5)
Weight, kg	73.1 (17.0)	77.4 (14.6)	68.7 (18.3)
Height, cm	168 (9.5)	175 (6.9)	162 (6.3)
BMI, kg/m^2^	25.8 (5.8)	25.2 (4.4)	26.4 (6.8)
Current smoker, *n* (%)	40 (10.9)	27 (14.5)	13 (7.1)
Current alcohol drinker, *n* (%)	146 (39.8)	91 (48.9)	55 (30.4)
Length of stay, days, median (IQR)	5 (3−8)	5 (3−7)	5 (3−9)
Living independently, *n* (%)	339 (90.9)	178 (92.7)	161 (89.0)
Risk of malnutrition, *n* (%)	130 (34.5)	70 (36.5)	60 (32.4)
Use of walking aid, *n* (%)	200 (53.3)	92 (48.2)	108 (58.7)
Self‐reported balance problems, *n* (%)	135 (36.4)	57 (30.0)	78 (43.1)
Immobile >1 week in previous 3 months, *n* (%)^a^	34 (14.3)	19 (15.7)	15 (12.8)
ADL dependent, *n* (%)	221 (59.2)	120 (62.5)	101 (55.8)
Number of medications, median (IQR)	8 (6–11)	8 (6–11)	8 (6–11)
Number of diseases, median (IQR)	3 (2–5)	3 (2–5)	3 (2–5)
Muscle mass and strength measures			
ALM/height^2^, kg/m^2^ b	7.05 (1.4)	7.64 (1.4)	6.48 (1.2)
ALM/BMI^b^	0.798 (0.23)	0.942 (0.21)	0.658 (0.15)
SMM, %^b^	36.4 (6.0)	39.1 (5.0)	33.7 (5.7)
SMI, kg/m^2^ b	9.16 (1.5)	9.70 (1.5)	8.64 (1.2)
Handgrip strength, kg	20.6 (9.8)	26.1 (9.9)	14.9 (5.6)

Note

All variables are presented as mean with standard deviation (*SD*) unless indicated otherwise. BMI: body mass index; IQR: interquartile range; ADL: activities of daily living; ALM: appendicular lean mass; SMM: skeletal muscle mass; SMI: skeletal muscle mass index.

a *n* = 238, females (*n* = 117), males (*n* = 121).

b *n* = 321, females (*n* = 163), males (*n* = 158).

### 3.2 Diagnostic criteria for sarcopenia

The prevalence rates of sarcopenia according to different applied diagnostic criteria, stratified by sex is shown in Table 2. Males had a higher prevalence rate of sarcopenia compared with females in all but one of the applied diagnostic criteria, that is, muscle strength as a single diagnostic criterion. Depending on the applied diagnostic criterion, the prevalence of sarcopenia in males ranged from 12.0%–75.9% in males and from 3.1%–75.3% in females. Based on the single measure‐based criterion using only muscle strength, 69.7% of male patients and 75.3% of female patients were considered sarcopenic. Based on the multiple measure‐based criteria using both measures of muscle mass and strength, prevalence ranged from 14.6%–75.9% in males and from 4.9%–24.5% in females.

**Table 2:** Diagnostic criteria of sarcopenia and prevalence of sarcopenia in older inpatients

First author or study	Diagnostic criteria	Cut‐off points		Prevalence, *N* (%)	
		Males	Females	Males (*N* = 158)	Females (*N* = 163)
Baumgartner, 1998	ALM/height^2^	≤7.26 kg/m^2^	≤5.45 kg/m^2^	71 (44.9)	31 (19.0)
Delmonico, 2007	ALM/height^2^	≤7.25 kg/m^2^	≤5.67 kg/m^2^	70 (44.3)	40 (24.5)
Kelly, 2009	ALM/height^2^	≤6.19 kg/m^2^	≤4.73 kg/m^2^	19 (12.0)	5 (3.1)
Janssen, 2002	(SMM/body mass) × 100%				
Class I		<37%	<28%	54 (34.2)	25 (15.3)
Class II		<31%	<22%	11 (7.0)	0 (0)
Janssen, 2004	SMI (SMM/height^2^)				
Moderate		≤10.75 kg/m^2^	≤6.75 kg/m^2^	120 (75.9)	8 (4.9)
Severe		≤8.50 kg/m^2^	≤5.75 kg/m^2^	36 (22.8)	0 (0)
Lauretani, 2003	Handgrip strength	<30.3 kg	<19.3 kg	133 (69.3)^a^	140 (75.3)^a^
EWGSOP, 2010	Total sarcopenic			120 (75.9)^b^	8 (4.9)^b^
	Subtotal sarcopenic based on gait speed ≤0.8 m/s			120 (75.9)	8 (4.9)
	Gait speed	≤0.8 m/s	≤0.8 m/s	N/A	N/A
	BIA: SMI (SM/height^2^)	≤10.75 kg/m^2^	≤6.75 kg/m^2^	120 (75.9)	8 (7.9)
	Subtotal sarcopenic based on gait speed >0.8 m/s			83 (52.5)	7 (4.3)
	Gait speed	>0.8 m/s	>0.8 m/s	N/A	N/A
	Handgrip strength	<30 kg	<20 kg	96 (60.8)	124 (76.1)
	BIA: SMI (SM/height^2^)	≤10.75 kg/m^2^	≤6.75 kg/m^2^	83 (86.5)	7 (5.7)^c^
IWGS, 2011	Total sarcopenic based on gait speed <1.0 m/s			70 (44.3)	40 (24.5)
	Gait speed	<1.0 m/s	<1.0 m/s	N/A	N/A
	ALM/height^2^	≤7.23 kg/m^2^	≤5.67 kg/m^2^	70 (44.3)	40 (24.5)
FNIH, 2014	Weakness and low lean mass			23 (14.6)	10 (6.13)
	Handgrip strength	<26 kg	<16 kg	74 (46.8)	87 (53.4)
	ALM/BMI	<0.789	<0.512	35 (22.2)	15 (9.2)

Note

ALM: appendicular lean muscle mass; SMM: skeletal muscle mass; SMI: skeletal muscle mass; EWGSOP: European Working Group on Sarcopenia in Older People; N/A: not available; BIA: bioelectrical impedance analyser; IWGS: International Working Group on Sarcopenia; FNIH: Foundation for the National Institutes of Health; BMI: body mass index;

a Males (*n* = 192), Females (*n* = 186).

b Prevalence of total sarcopenia based on BIA measurement which were performed in the inpatients.

Proportion of the number of cases with handgrip strength <30 kg.

c Proportion of the number of cases with handgrip strength <20 kg.

**Figure 1:**
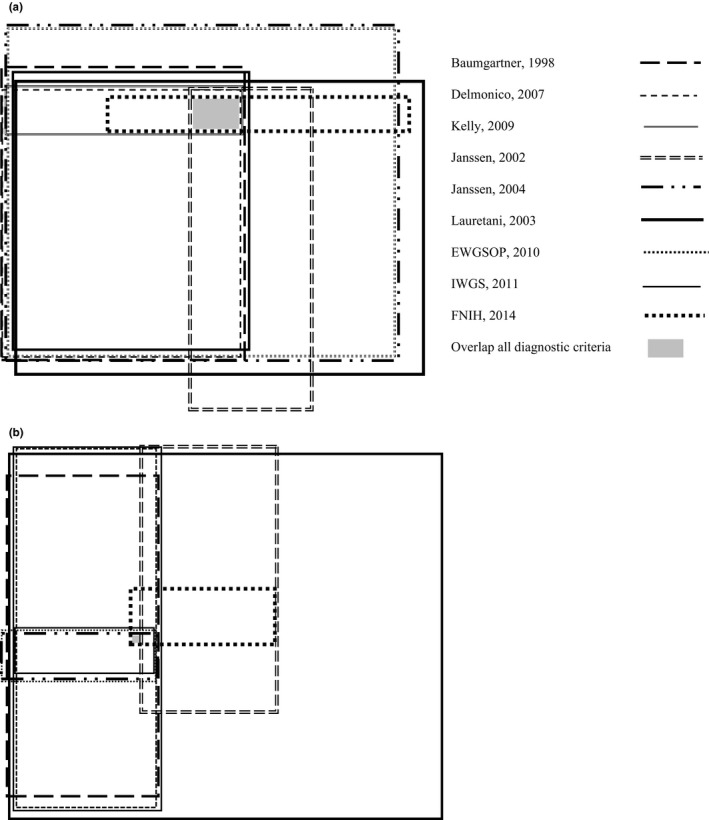
Number of geriatric inpatients identified as having sarcopenia according to various diagnostic criteria. (a) Male inpatients, *n* = 158; percent of total: Baumgartner: 44.9%, Delmonico: 44.3%, Kelly: 12.0%, Janssen, 2002: 34.2%, Janssen, 2004: 75.9%, Lauretani: 65.8%, EWGSOP: 75.9%, IWGS: 44.3%, FNIH: 14.6%. Total overlap: 5 (3.2%) male inpatients. (b) Female inpatients, *n* = 163; percent of total: Baumgartner: 19.0%, Delmonico: 24.5%, Kelly: 3.1%, Janssen, 2002: 15.3%, Janssen, 2004: 4.9%, Lauretani: 75.5%, EWGSOP: 4.9%, IWGS: 24.5%, FNIH: 6.1%. Total overlap: 1 (0.6%) female inpatients

Figure 1 shows the overlap between different diagnostic criteria stratified by sex. Five males (3.2%) and one (0.6%) female patient were classified as sarcopenic according to all applied diagnostic criteria. Most inpatients with sarcopenia according to diagnostic criteria based on low muscle mass were also sarcopenic according to diagnostic criteria based on low muscle strength.

## 4 DISCUSSION

This is the first study investigating the prevalence of sarcopenia according to nine commonly used diagnostic criteria for sarcopenia in a hospitalized older patient population. Among older inpatients, prevalence rates of sarcopenia were high, especially in male inpatients and varied substantially depending on the applied diagnostic criteria.

The huge difference in prevalence of sarcopenia found in our inpatient population was due to the varying combination of used diagnostic criteria. Multiple measure‐based criteria, combining measures of muscle mass, muscle strength and/or physical performance (Cruz‐Jentoft et al., 2010; Fielding et al., 2011; Studenski et al., 2014), showed lower prevalence of sarcopenia compared with the single measure‐based diagnostic criteria. This finding is consistent with results in community‐dwelling older individuals (Pagotto & Silveira, 2014). Inpatients who had low muscle strength did not always have low muscle mass, as loss of muscle strength generally occurs at a faster rate compared with loss of muscle mass (Goodpaster et al., 2006). The discrepancy between muscle mass and muscle strength at individual level may explain why the single diagnostic criterion of muscle strength gave a high prevalence of sarcopenia in both sexes.

Interestingly, this study has highlighted a large difference in the prevalence of sarcopenia between male and female inpatients. Using the EWGSOP diagnostic criteria for sarcopenia, 75.9% of males and 4.9% of females were diagnosed as sarcopenic. In contrast, the GLISTEN study reported no sex differences in prevalence of sarcopenia in hospitalized older patients using the EWGSOP criteria (Bianchi et al., 2017). Using varying diagnostic criteria, previous studies reported relatively consistent prevalence of sarcopenia between sexes in older inpatients (Bianchi et al., 2016; Perna et al., 2017; Sousa, Guerra, Fonseca, Pichel, & Amaral, 2015; Vetrano et al., 2014). Unlike the present study, the GLISTEN study also used physical performance as a diagnostic measure (4 m gait speed test) to diagnose sarcopenia. If gait speed was unavailable, a reported ADL score was used to estimate normal or abnormal gait speed, which was the case in more than 40% individuals (Bianchi et al., 2017). Inability to perform physical performance measures is a common hurdle in hospitalized older patients in applying certain diagnostic criteria for sarcopenia (Cruz‐Jentoft et al., 2010; Fielding et al., 2011; Studenski et al., 2014). We did not replace the physical performance measure with an ADL score as this has not been validated yet in hospitalized older patients.

Diagnostic criteria for sarcopenia included in this study have been designed to be applied to the general population and not for an inpatient population. To the best of our knowledge, no specific diagnostic criteria have been defined yet for inpatients, for example, taking the influence of acute disease into account. Also, it has not yet been defined which healthcare professional is responsible measuring for body composition in clinical practice. A role for nursing in diagnosing and intervening in sarcopenia would be obvious, but protocols have yet to be established. Future longitudinal studies should focus on the need of adjusted diagnostic criteria in inpatients, definition of the proper windows of assessment and the role of individual healthcare professionals in diagnosing sarcopenia.

### 4.1 Strengths and limitations

This study included a large number of patients, who were acutely and subsequently admitted to various wards of the hospital, minimizing selection bias. Inability to obtain measurements of gait speed in the acute phase prevented the proper use of diagnostic criteria based on physical performance. The measurements of body composition using BIA may have been affected by hydration status, influencing the ratio of muscle mass/fat mass.

## 5 CONCLUSION

In hospitalized older inpatients, acutely and electively admitted to different wards in an academic hospital, prevalence of sarcopenia is highly dependent on the applied diagnostic criteria and higher in males compared with females. Future research should focus on specific diagnostic criteria for inpatients as those currently used often include measures of physical performance, which can hardly be obtained at hospital admission due to severe illness.

## CONFLICTS OF INTEREST

None to report.

## AUTHORS CONTRIBUTIONS

AB, EMR, SV, CGMM, ABM: Made substantial contributions to conception and design, or acquisition of data, or analysis and interpretation of data; AB, EMR, CSLT, JVA, CGMM, ABM: Involved in drafting the manuscript or revising it critically for important intellectual content; AB, EMR, CSLT, JVA, SV, CGMM, ABM: Given final approval of the version to be published. Each author should have participated sufficiently in the work to take public responsibility for appropriate portions of the content; AB, EMR, CSLT, JVA, SV, CGMM, ABM: Agreed to be accountable for all aspects of the work in ensuring that questions related to the accuracy or integrity of any part of the work are appropriately investigated and resolved.
